# Barriers in the implementation of interprofessional continuing education programs – a qualitative study from Germany

**DOI:** 10.1186/1472-6920-14-227

**Published:** 2014-10-21

**Authors:** Sibel V Altin, Ralf Tebest, Sibylle Kautz-Freimuth, Marcus Redaelli, Stephanie Stock

**Affiliations:** Institute for Health Economics and Clinical Epidemiology, University Hospital of Cologne, Gleuelerstr. 176-178, 50935 Cologne, Germany; Institute of General Practice, Medical Faculty, Heinrich-Heine University, Moorenstraße 5, 40225 Düsseldorf, Germany

**Keywords:** Medical education, Interprofessional continuing education, Needs assessment, Health policy implications

## Abstract

**Background:**

Insufficient communication and coordination is one of the most problematic issues in German health care delivery leading to detrimental effects on health care outcomes. As a consequence interprofessional continuing education (CIPE) is gathering momentum in German health policy and health care practice aiming to enhance service quality and patient safety. Nevertheless, there is limited evidence on the course of implementation and the perceived effectiveness/acceptance of CIPE in German health care. This paper describes the objectives and formal characteristics of CIPE trainings and maps important determinants influencing the success of CIPE implementation from the perspective of providers offering CIPE trainings for German health care professionals.

**Methods:**

Forty-nine training institutions offering CIPE for health care professionals were identified by a structured web search including the websites of German medical education associations and public/private training institutions. Directors and managers of the identified institutions were invited to participate in a semi-structured interview. The interview guideline was developed using the SPSS method by Helferich and colleges. Interviews were analyzed using the summarizing content analysis developed by Mayring resulting in a paradigm that contextualizes hindering factors regarding the implementation of CIPE in the German health care system.

**Results:**

Overall, 19 of the identified institutions agreed to participate with one director/manager per institution resulting in a response rate of almost 38.8%. The included institutions offer n = 85 CIPE trainings for health care professionals. Trainings offered mainly address the enhancement of domain, social and personal competencies of the participating health care professionals and follow three main objectives comprising better care of severely ill patients, improvement of patient safety by sustained risk management as well as a more patient centered care. Implementation of CIPE in Germany is influenced by various hindering factors mostly coming from systemic (missing incentives), behavioral (hierarchy problems) and methodological (limited quality assurance) factors.

**Conclusion:**

CIPE is an evolving concept in the German health care system. There are various difficulties that impede a successful implementation of CIPE and might be mitigated by specific health policy interventions such as mandatory CIPE participation of health care professionals and comprehensive pre-license interprofessional education.

**Electronic supplementary material:**

The online version of this article (doi:10.1186/1472-6920-14-227) contains supplementary material, which is available to authorized users.

## Background

Despite efforts to integrate care better, there are still strong barriers between inpatient and outpatient care in the German health care system, which lead to fragmentation in chronic care and unsatisfying patient outcomes [1, 2]. As a consequence, the German Ministry of Health followed the internationally evolving concept of interprofessional care aiming to strengthen health care quality and patient safety by legislating more and more quality assurance measures for in- and outpatient care [3]. Consequently, health care organizations reacted to this approach by investing in human resource development [4]. The overarching objective is to increase interprofessional competencies of the health care workforce to enhance cooperation between health professionals and allow for better communication with patients. The latter objective refers to the ongoing movement to transform health care organizations to health literacy friendly ones that address informational needs of patients and consider their values and preferences [5]. Whereas better cooperation between health professionals is achieved by applying continuing interprofessional education (CIPE) as a preferred model to implement interprofessional education (IPE).

CIPE occurs after qualification or licensure and is defined as the process in which two or more health and/or social care professions learn with, from, and about each other to improve collaboration and subsequently quality of care [6]. In most cases CIPE is offered in workshops and seminars aiming to improve the delivery of interprofessional care [7]. In the German context, CIPE trainings are used to enhance action competence by educating health care professionals in domain/subject as well as social and personal competence. The former describes the ability to acquire subject-specific knowledge whereas social competence denotes the ability to shape relationships by interacting with others in a rational and conscientious way. Personal competence embodies both cognitive and social competences and includes personal characteristics such as critical abilities, self-confidence, reliability and responsibility [8].

Although the implementation of CIPE in Germany evolved in recent years, there is limited evidence on the course of this process and the perceived effectiveness/acceptance of CIPE. Several German papers describe the general necessity and the theoretical foundation of interprofessional care offering limited insights in its actual implementation [4, 9, 10].

In order to fill this gap, we performed a qualitative study to describe the scope of CIPE implementation in German medical education and map important determinants influencing its implementation and application.

## Methods

### Study

We carried out a qualitative study performing semi-structured telephone based expert interviews among directors/managers of German medical education institutions.

### Development of semi-structured interview guide

The semi-structured interview manual was developed by a systematic literature review on the status quo of continuing interprofessional education (CIPE) in Germany. We particularly considered the supply structure/density, accessibility and design of offered courses as well as regulative and policy based factors in continuing medical education (CME) and interprofessional care. By performing the review, we identified possible barriers and facilitators in CIPE implementation in Germany. These information were used as a basis to develop the semi-structured interview guideline that was incorporated using the SPSS method by Helferich and colleagues [11]. In accordance with the method we determined the interview purpose and collected sensitive issues by performing a brain storming with an interprofessional team (physician, nurse scientist, sociologist), applying the preamble oriented pyramid discussion based on the results of the review [12]. The final semi-structured interview covers two domains: characteristics of provided CIPE trainings (design, access, structure, topics) (12 questions) and experience with CIPE trainings (6 questions). The following pilot-tests (N = 5 CME executives) did not substantiate changes in content but resulted in minor refinements in the wording of some questions.

### Interview sample

We identified 49 training institutions offering CIPE training by performing a structured web search including homepages of German medical education associations and public/private training institutions. The structured web search was performed by utilizing certain search strategies and key words presented in Additional file 1: Table S1. In accordance to the definition of CIPE medical education institutions were eligible for inclusion when delivering (continuing) education programs in which two or more health and/or social professions learn with, from and about each other. Institute directors/managers were contacted electronically (email) detailing the study purpose and inviting them to participate in a telephone interview. Overall, 19 of the identified institutions agreed to participate resulting in a response rate of almost 38.8%. We recruited one executive per site (N = 19) to participate in the semi-structured telephone interview. By targeting executives, we intended to gain insight into their extensive experiences in CIPE planning and implementation. Overall, the interviews included 18 items and had an average duration of 45 minutes. All interviews were gathered in a period of two months between September and November 2012.

Our qualitative study involved semi-structured interviews and was carried out in accordance with the Declaration of Helsinki. The potential for risk to the interview participants was reduced to a minimum by obtaining informed consent prior to data collection and de-identifying the interviews. All participants were assured confidentiality. Ethical approval from the university hospital of Cologne was not required for this type of qualitative study.

### Data analysis

Interviews were tape-recorded, transcribed verbatim, and analyzed using the summarizing content analysis developed by Mayring [13]. The method seeks to reduce the material to its essential content in a systematic manner by following a four-step sequence model resulting in a summary of the main statements in a paradigm. A paradigm was developed for hindering factors in CIPE training implementation in the German health care context. Therefore, two researchers divided the interview statements into meaning-carrying units and independently categorized these to a paradigm. In order to enable researcher triangulation we made sure that only one researcher participated in the development of the interview guide enabling objectivity [14]. Results were included only when agreement was achieved between both researchers regarding the interpretation of the data. In order to describe demographic features and the supply structure and density of all identified CIPE formats we applied descriptive statistics.

## Results

Overall, 19 directors/managers (response rate: 38.8%) participated in the semi-structured expert interviews conducted via telephone. The majority of participants were females (63.2%; n = 12) and had an educational and vocational background in nursing (52.6%; n = 10) as depicted in Table 1. Most educational institutions are owned by public institutions and are affiliated with hospitals, which use CIPE trainings as a tool for human resource development. More than every second institution offers CIPE trainings for internal and external staff, which means the seminars are accessible for hospital staff as well as interested parties from other healthcare organizations. Though the implementation of CIPE trainings has increased since the turn of the millennium, interprofessional continuing medical education is still a relatively new phenomenon in Germany.

**Table 1:** **Interview sample characteristics**

	Sample characteristics	
	N	%
	19	
*Interviewee*		
Female	12	63.2
Male	7	36.8
*Vocational background*		
Nursing scientists/practitioners	10	52.6
Physicians	1	5.3
Others (pedagogues)	8	42.1
Executive position	19	100
*Institutions*		
Private education side	3	15.8
Public education side	14	73.7
Non-profit education side	2	10.5
Affilitation to hospitals	14	73.7
Internal provider	7	36.8
External provider	1	5.3
Internal and external provider	11	57.9
*Offering CIPE since*		
1970-1999	4	21.1
2000-2012	15	78.9

### Characteristics of CIPE programs in Germany

By means of the key informant interviews we identified N = 85 CIPE trainings for health care professionals. These aim to enhance the domain, social or personal competences of the participants. The majority of courses (42.4%, n = 36) have the objective to increase domain related competency. Overall, 79% (n = 15) of the interviewees also reported that although their CIPE trainings are offered to all health care professionals including physicians, participants are mainly nurses.

When considering the available CIPE trainings, a wide variety of issues is noted. Among the 85 trainings described by our informants those addressing domain competence mainly focus on the clinical field, providing trainings related to certain health conditions/diseases or to therapy management, often associated with end of life care. The latter also applies to programs that aim to enhance social competencies such as trainings in terminal care. CIPE trainings offered to enhance social competencies mainly focus on patient related factors in health care delivery. Main objectives include an appropriate communication (42.3%; n = 11) with and counseling (15.4%; n = 4) of severely ill patients and their relatives. The identified CIPE trainings that aim to enable the development of personal competence focus on supporting healthcare professionals to better cope with the challenges of their daily vocational activities (stress and error management, leadership) and by training them to better cooperate and coordinate in a team as shown in Table 2.

**Table 2:** **Number of identified CIPE programs in accordance to competence type**

	CIPE programs	
	N	%
***Domain competence***		
Wound management	8	22,2
Palliative care	6	16,7
Pain management	5	13,9
Dementia care	5	13,9
Reanimation	4	11,1
Hygiene management	4	11,1
Drug safety and management	4	11,1
***Personal competence***		
Team competence training	7	30,4
Stress management	6	26,1
Leadership competences	4	17,4
Communication and coordination	3	13,0
Error management culture	3	13,0
***Social competence***		
Communication with patients and relatives	11	42,3
Counseling of patients and their relatives	4	15,4
Terminal care	4	15,4
Caring for patients with dementia	3	11,5
Dealing with aggressive patients	2	7,7
Intercultural communication	2	7,7

Note: Total number of CIPE offers N = 85; Percentages are calculated according to the total number of CIPE offers per competence type: domain competence offers N = 36; personal competence offers N = 23; social competence offers N = 26.

### Experiences with CIPE application

**Figure 1:**
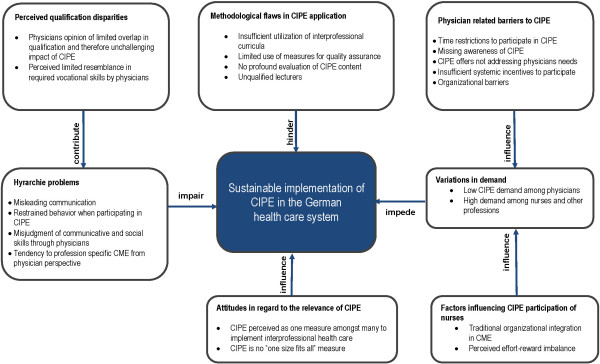
**Paradigm on the barriers of CIPE application in Germany (N = 19).**

By conducting semi-structured interviews we aimed to elicit information on two key aspects on CIPE: the characteristics of available trainings (design, access, structure, topics) and previous experiences (barriers) in the provision of CIPE trainings. This comprehensive approach enables to describe the general context of the topic and develop a paradigm as shown in Figure 1.

### Barriers for CIPE implementation and application in German healthcare

The analysis of the interviews resulted in the identification of four barrier types that influence the implementation of CIPE in the German health care: systemic (incentive-based), behavioral, attitudinal and methodological barriers. Definitions and classifications of all barrier types are displayed in Table 3. The results for each barrier type are presented with illustrative quotes from the interviewees (IN/number).

**Table 3:** **Barriers influencing the implementation of CIPE in German health care**

Category	Definition	Subcategory	Selection of illustrations
Sytemic barriers	Hindering factors coming from insufficient CIPE delivery structures and missing incentives to participate	Variation in CIPE demand due to missing physician related incentives to participate and sufficient nurse-related incentives	*“Most physicians in the hospital context need very specific educational courses most educational sites cannot provide”. (IN7)*
		Insufficient utilization of public relation measures to familiarize new target groups (professions) with CIPE	*“Physicians mainly participate in CIPE trainings when they can receive CME credits”. (IN2)*
			*“What we often hear is, “Oh, you also have a program for us?” That’s irritating because we offer our CIPE program for years now and we tried to find adequate multiplicators to distribute the information to physicians”. (IN6)*
Behavioral barriers	Hindering factors coming from differing professional socialization processes	Hyrarchie problems between professions participating in CIPE	*“Well, especially physicians tend to behave very bored when visiting CIPE trainings. Often they react harsh saying that this is a waste of time”. (IN10)*
		Misleading communication patterns and structures due to hierarchical structures in health care organizations	*“What our lecturers experience very often is that physicians admit that they often rely on nurses when it comes to communication with patients. I think that this might be one reason why they do not feel responsible for these things”. (IN17)*
		Differing attitudes in regard to the relevance of CIPE between health care professions	
Methodological barriers	Hindering factors coming from an insufficient utilization of quality assurance measures among CIPE providers	Insufficient utilization of curricula and standardized evaluation procedures	*“There is much flexibility in the design of continuing medical education leading to structural and quality assurance problems”. (IN19)*
		Qualification gap among CIPE lecturers	*“By now, we do not use specific requirements when it comes to recruit lecturers for our CIPE offers. We are more than satisfied if we receive applications of lecturers who worked with various professional groups”. (IN15)*
Attitudinal barriers	Hindering factors associated with general attitudes regarding the role, relevance and effectiveness of CIPE in medical education	Perception of CIPE as no “one size fits all” strategy for the realization of interprofessional health care delivery	*“What we are doing is very important and we experience that it helps a lot to find solutions to problems in health care delivery. But on the other hand what we do is a drop in the bucket. CIPE is one link in the chain. It requires more efforts in the education of health professions”. (IN12)*
		CIPE as one measure amongst many	

### General attitudes regarding the role and relevance of CIPE

Our analysis yields the finding that general attitudes regarding the role, relevance and effectiveness of CIPE in medical education have a significant impact on the application of CIPE. In this regard, the interviewees recommend to not misjudge the effect of CIPE trainings as a universal solution or a one size fits all remedy to improve health care delivery.

One interviewee notes:

*“What we are doing is very important and we experience that it helps a lot to find solutions to problems in health care delivery. But on the other hand what we do is a drop in the bucket. CIPE is one link in the chain. It requires more efforts in the education of health professions”. (IN12)*

Consequently, interviewees demand the provision of interprofessional trainings already in health care education and pre-license medical training to raise awareness for and facilitate the idea of interprofessional care among novice healthcare professionals. The participants hope to achieve a sustainable “added value” and attitude change through the integration of interprofessional practice into the vocational socialization of health care professionls.

### Methodological barriers - quality assurance issues in CIPE application

As shown in Figure 1, respondents report that there are methodological flaws that often hinder a comprehensive assessment of CIPE training effects on health care delivery. These flaws are mainly due to insufficient utilization of scientific methods when designing and applying a CIPE trainings.

According to the interviewees interviewed, *“there is much flexibility in the design of continuing medical education leading to structural and quality assurance problems*” *(IN19).*

Most respondents report that they are not aware of valid guidelines on the development of curricula for CIPE trainings, which might result in flaws in the evaluation of the courses. It was noted that 52.9% (n = 16) of the educational institutions do not use curricula to determine content and structure of CIPE trainings. Interviewees also report that there is no supplementary formal qualification for interprofessional health care education that considers the vocational needs of all involved professions. They affirm that training requirements might not meet informational needs of all professions resulting in dissatisfaction among participants. Further, the evaluation processes used by almost all of the respondents mainly focus on the participants satisfaction with the lecturers performance not necessarily recognizing the course content. This leads to insufficient evaluation of the results, most of which are consequently of limited significance.

### Systemic barriers - variations in demand on CIPE trainings

Interviewees report negative experiences concerning the practical implementation of CIPE trainings due to an imbalance of the participating health care professions as depicted in Figure 1. Therefore, 76.5% (n = 13) declare that physicians rarely participate in CIPE trainings, whereas nurses and other health professionals take part very often. This imbalance is seen as one of the major problems of CIPE implementation, because a transition in health care delivery is only possible when all professions take part, including physicians who play a central role in routine care. Respondents analyze this problem from different perspectives resulting in differing explanations for the underlying reasons. Some of them assume that CIPE courses might not meet the informational needs of physicians. Thus, they propose a more comprehensive needs assessment. In this regard one interviewee reports:

*“Most physicians in the hospital context need very specific educational courses most educational sites cannot provide”. (IN7)*

Others suppose that the incentives to participate in such courses are insufficient for physicians with one interviewee assuming that *“physicians mainly participate in CIPE trainings when they can receive CME credits”. (IN2)*

For others the imbalance is based on the organizational structures of hospitals, because continuing education was originally organized by nursing directors and aligned to the educational needs of nurses. Although in the past this institutional setting helped to develop a training culture in the nursing field, today it represents a barrier for physicians to participate in CIPE trainings. Therefore, interviewees recommend to enhance public relations in order to raise awareness among physicians. In this regard, one interviewee notes a follows:

*“What we often hear is, “Oh, you also have a program for us?” That’s irritating because we offer our CIPE program for years now and we tried to find adequate multiplicators to distribute the information to physicians”.(IN6)*

Other participants state that a lack of time is responsible for the limited participation of physicians, emphasizing that physicians are often not exempted from work to participate in CIPE trainings.

### Behavioral barriers- communication and hierarchy problems

Interviewees report that differences in vocational qualification among heterogeneous groups participating in CIPE trainings sometimes lead to misunderstandings due to differences in communication strategies as shown in Figure 1. Participants emphasize that physicians often believe they already know the task profile, skills and communication types of other healthcare professionals causing them to assume that they do not need CIPE trainings to enhance their communication or coordination skills. Other interviewees emphasize that physicians often feel subchallenged when participating in CIPE trainings developed to enhance domain competence.

According to that, one interviewee critically reports:

*“Well, especially physicians tend to behave very bored when visiting CIPE trainings. Often they react harsh saying that this is a waste of time”. (IN10)*

Interviewees also report that physicians participating in CIPE trainings often state they do not feel responsible for communication and defer to nurses. Further, the participants affirm that physicians often do have restraints to participate in CIPE Programs together with nurses whereas latter behave reserved resulting in limited engagement in discussions due to self-esteem problems. Respondents conclude that these attitudes may indicate a hierarchy problem resulting from perceived qualification disparities and miscommunication that affects social action patterns.

All in all, respondents agree that there is no data on how to increase motivation of physicians to participate in CIPE trainings. As a consequence, CIPE training providers can only guess why difficulties in motivating physicians to engage in CIPE are occurring. The consequence is a persistent lack of effective interventions. They conclude that interprofessional continuing education presupposes an interprofessional group of participants to reach its aim.

## Discussion

Our intention was to describe the scope of CIPE training implementation in German medical education and map important determinants influencing its implementation and application.

In our analysis, we determined characteristics of CIPE trainings and developed a paradigm of important determinants that influence the implementation and perceived effectiveness/acceptance of CIPE in Germany.

According to our semi-structured interviews CIPE is a relatively new but evolving concept in German CME, especially offered by academies in inpatient facilities and used for human resource development. The offered CIPE courses address domain, social and personal competencies of the healthcare work force and follow three main objectives: 1. better care of severely ill patients, 2. improvement of patient safety by sustained risk management and 3. a more patient centered care. In recent years, all these issues have become integral components of German health care policy fostered through several courses of action [2]. CIPE is used as an instrument to transfer best practice findings into heath care delivery.

Although CIPE trainings address a variety of relevant issues in health care delivery, some significant aspects are still missing. Therefore the German Advisory Council on the Assessment of Developments in the Healthcare System especially recommended the development of interprofessional education (IPE) formats for health professionals working in the fields of pediatrics, oncology and neurology [1]. Our analysis demonstrates that CIPE trainings in these areas are scarce.

Furthermore, we identified main barriers of CIPE implementation in Germany. Interviewees recommend to refrain from believing that CIPE training alone is the ultimate solution to implement interprofessional care sustainably and instead suggest to add aspects of interprofessional education (IPE) in pre-license education to facilitate its implementation. This requirement is consistent with the recommendations of the Advisory Board of the Ministry of Health [1]. Nevertheless pre-license trainings in IPE are very scarce in Germany [15, 16] and international reviews also report that a substantial amount of IPE occurs in CME rather than in a pre-license context [6, 17].

We determined several factors of CIPE implementation that are perceived as serious barriers. These occur due to methodological problems when developing, implementing and assessing CIPE programs, as well as practical application problems. There are flaws in regard to curriculum development, lecturer qualification and CIPE training evaluation. A review of the CME literature yields several theories and methods for developing and assessing CIPE programs thoroughly [18, 19]. However, our results demonstrate that they are rarely used among the German educational sites participating in our qualitative study. Furthermore, appropriate training for lecturers of CIPE is scarce. On balance, the professionalization process in CIPE implementation in Germany is not yet finished.

Another important barrier we identified is the imbalance in participation due to systemic, (incentive-related) organizational, communication based factors and hierarchy problems. By now, data on the participation of different professions in CIPE trainings is scarce due to the limited availability of studies dealing with CIPE implementation or evaluation in the German health care context. Therefore, our results regarding the rare participation of physicians in CIPE training and the potential reasons for this imbalance can be used as a basis for the development of novel health policy interventions in order to facilitate CIPE implementation in Germany. There are many ways to set incentives for physicians to participate in CIPE trainings. For instance, one might strengthen the needs analysis performed due to the development of CIPE offers in order to better respond to physicians needs. Further, it may be helpful to introduce a mandatory participation in CIPE courses for all health professions. Similar regulations were implemented in 2011 when the revision of the German Infection Protection Act (Infektionsschutzgesetz 2011) came into effect, specifying mandatory participation in hygiene management courses for health care professionals. Another viable option could be to offer more certified CIPE courses to increase the incentive for physicians to participate. In Germany, continuing medical education for physicians is mandatory. According to the Health Modernization Act “Gesundheitsmodernisierungsgesetz (GMG)” which came into force in January 2004, every medical specialist must attend CME trainings equivalent to 250 CME points within five years to receive a continuing education certificate from the German Medical Chamber. Subsequently, physicians have a greater incentive to attend certified CME trainings to fulfil the obligation.

Since educational and vocational socialization have an important impact on the social identity of professions by enhancing their recognition of a group membership as a part of their individual self-concept [20] interventions enhancing interprofessional experiences as a component of vocational socialization could also help reducing the profession related variations in CIPE participation. In this regard, the development and comprehensive implementation of IPE in pre-license education of health care professionals could also help to facilitate the IPE approach in vocational socialization leading to a spirit of openness by reducing hierarchy problems [7, 21, 22]. German inpatient healthcare organizations are particularly perceived to be associated with hierarchy related attitudes, which result in serious communication problems between nurses and physicians [23–25]. In this regard, it can be assumed that the distinct illustration of role barriers (hierarchy problem) are due to a large extent of respondents working in hospital based educational sites. Nevertheless, pre-license IPE would require extensive research on the determinants of faculty development to better understand what faculties need to do to support IPE [26].

Our study has some limitations that need to be discussed. First, we identified our sample by carrying out a structured web based search of educational institutions providing CIPE trainings. It may be that we did not locate all providers of CIPE trainings in Germany. Moreover, it has to be considered that the majority of our sample works at educational institutions attached to hospitals, where hierarchy problems might be more present. Further, it has to be considered that the majority of interviewees had a nursing background and only one interviewee was a physician. Therefore, statements regarding possible role barriers might also be influenced by vocational views of nurses. Nevertheless, barriers reported by our sample do probably also apply to other non-medical professions [27].

## Conclusions

A successful application of CIPE trainings in Germany is influenced by various determinants. Difficulties in achieving a balanced proportion of participating professions seem to arise from systemic, behavioral and methodological factors. These may be influenced by more in depth needs analysis on CIPE training design and additional health policy support. Further evidence and critical appraisal on the status quo of CIPE such as the evidence presented here is required.

## Authors’ information

All authors are affiliated to the Institute for Health Economics and Clinical Epidemiology, University Hospital of Cologne and primarily deal with health systems and outcomes research focusing on chronic care and disease management. Mrs. Prof. Dr. med. Stephanie Stock is the chairwoman of the German Health Literacy Network and coordinates the network activities in Germany.

## Electronic supplementary material

Additional file 1: Table S1: Search strategies for the structured web search. (DOCX 12 KB)

## Abbreviations

CIPE: Interprofessional continuing education
CME: Continuing medical education
IPE: Interprofessional education.
