# Supplementary material for: Barriers in the implementation of interprofessional continuing education programs – a qualitative study from Germany
Source: BMC Med Educ. 2014 Oct 21;14:227. doi: 10.1186/1472-6920-14-227 (PMC4288664; doi:10.1186/1472-6920-14-227)
Supplement: Supplementary file 1 — Additional file 1: Table S1: Search strategies for the structured web search. (DOCX 12 KB) [file 12909_2014_1049_MOESM1_ESM.docx]

**Additional file1: Table S1. Search strategies for the structured web search**

| **Search strategies** | **relevant hits** |
| --- | --- |
| „interprofessional“ OR „continuing education“ OR „health care“ | 6 |
| „interprofessionality“ OR „health care professionals“ | 4 |
| „continuing education“ OR „health care“ | 6 |
| „continuing education“ OR „health care“ OR „Germany” | 5 |
| „continuing education“ OR „education“ OR „medical education site“ OR „health care“ | 2 |
| „interprofessional“ OR „communication“ | 5 |
| „continuing education“ OR „health care“ OR „physician + nurse + therapist“ | 5 |
| „education“ OR „educational sites“ OR „health care“ | 4 |
| „education“ OR „health care organization“ OR „rehabilitation“ | 8 |
| „education sites“ OR „academy“ OR „health care“AND “interprofessional | 4 |
| **Sum of hits** | 54 |
